# Phase I study of niraparib with radiotherapy for treatment of metastatic and locally advanced invasive carcinoma of the cervix (NIVIX)

**DOI:** 10.3389/fonc.2026.1677424

**Published:** 2026-03-09

**Authors:** Piyush Pathak, Eunji Jo, Susan Hilsenbeck, Matthew L. Anderson, Alfredo Echeverria, Shelly Sharma, Jan Sunde, Tracilyn Hall, Anthony Costales, Claire Hoppenot, Michelle Ludwig

**Affiliations:** 1 Department of Radiation Oncology, Baylor College of Medicine, Houston, TX, United States; 2 Department of Biostatistics, Baylor College of Medicine, Houston, TX, United States; 3 Department of Obstetrics and Gynecology, University of South Florida (USF) Morsani College of Medicine, Tampa, FL, United States; 4 Department of Obstetrics and Gynecology, Baylor College of Medicine, Houston, TX, United States

**Keywords:** brachytherapy, cervix cancer, niraparib, PARP inhibitor, radiotherapy

## Abstract

**Introduction:**

This Phase I study evaluated the safety and tolerability of concurrent niraparib, an oral poly(ADP-ribose) polymerase (PARP) 1/2 inhibitor, with definitive radiotherapy in women with locally advanced or platinum-sensitive metastatic cervical cancer. Dose escalation followed a Bayesian Optimal Interval (BOIN) design with a target dose-limiting toxicity (DLT) rate of 30%.

**Methods:**

Women ≥18 years with FIGO stage IIIC2 (bulky nodes >1 cm) or stage IV cervical cancer demonstrating response to platinum-based induction chemotherapy were eligible. Niraparib 100 mg was administered daily concurrent with pelvic radiotherapy (45–57.5 Gy via IMRT with nodal boosts) and brachytherapy, continuing 28 days post-radiation. Per the BOIN design, dose escalation occurred if the observed DLT rate fell below the pre-specified boundary of 0.2365; de-escalation occurred if it exceeded 0.3585. DLTs were defined per protocol as grade ≥4 non-hematologic toxicity, grade 4 thrombocytopenia ≥14 days, grade 3–4 thrombocytopenia with bleeding, or toxicity causing >28-day niraparib delay or >2-day radiation delay.

**Results:**

Four patients were enrolled at the 100 mg dose level before study closure due to slow accrual and anticipated changes in standard of care. Two patients (NVX-1, NVX-4) completed concurrent niraparib without protocol-defined DLT. Two patients (NVX-2, NVX-3) discontinued niraparib due to treatment-limiting grade 2 thrombocytopenia (CTCAE v4.0; nadirs 71,000/μL and 53,000/μL); these events did not meet protocol DLT criteria, and no bleeding occurred. The observed DLT rate of 0/4 (0%) fell below the escalation boundary; however, the study closed before dose escalation. Both patients who discontinued had baseline platelet counts <150,000/μL and significant comorbidities (HIV/cirrhosis; suspected chemotherapy-related marrow injury), whereas the two who completed therapy had counts >250,000/μL. All patients completed radiotherapy and achieved local disease control at minimum 12-month follow-up.

**Conclusion:**

Concurrent niraparib 100 mg with pelvic radiotherapy was feasible without protocol-defined DLTs. Treatment-limiting grade 2 thrombocytopenia occurred in patients with identifiable risk factors including low baseline platelet counts, hepatic dysfunction, and low body weight. These hypothesis-generating findings support further investigation with attention to patient selection criteria in future trials combining PARP inhibitors with radiotherapy.

## 1 Introduction

Invasive carcinoma of the uterine cervix accounts for approximately 14,000 new cases and 4,300 deaths each year in the United States, with a disproportionate impact on disadvantaged communities with limited access to screening and treatment (1). Most women with cervical cancer in the U.S. present with metastatic or locally advanced disease (LACC) requiring chemoradiation (2, 3). The integration of concurrent chemotherapy with radiation represents a significant advancement, improving 5-year overall survival by 6% compared to radiation alone (from 60% to 66%; HR 0.81, 95% CI 0.71-0.91) in a systematic review and meta-analysis of 13 studies (4). However, this improvement comes at the cost of increased toxicity, particularly hematologic and gastrointestinal adverse effects, which can compromise treatment completion. Adherence rates as low as 49% have been reported in the literature (5).

Despite advances in combination therapy, the prognosis for patients with metastatic or LACC remains poor, with 5-year survival rates of only 18% and 61% respectively (2). Recent studies evaluating neoadjuvant chemotherapy and concurrent immunotherapy approaches have shown improved outcomes (6, 7), and are increasingly utilized in clinical practice. However, these studies include a large proportion of early-stage patients and long-term outcomes data are not yet available. New therapeutic strategies are therefore still necessary to improve outcomes for these patients.

Poly(ADP-ribose) polymerase (PARP) inhibitors have emerged as a promising class of agents that exploit DNA repair deficiencies in cancer cells. By preventing the repair of single-strand DNA breaks, these drugs lead to the accumulation of double-strand breaks during replication, ultimately causing genomic instability and cell death (8). While particularly effective in tumors with homologous recombination deficiency, such as BRCA-mutated breast and ovarian cancers (9), emerging evidence suggests broader applications, especially in combination with DNA-damaging therapies like radiation.

The oral PARP inhibitor niraparib has demonstrated particular promise in gynecologic malignancies. Initially approved for maintenance therapy in recurrent ovarian cancer based on the NOVA trial, which showed significant improvement in progression-free survival regardless of BRCA mutation status (10), subsequent studies have expanded its utility. The PRIMA trial demonstrated marked benefit in newly diagnosed patients (21.9 vs 10.4 months; HR 0.43; P<0.001) (11), establishing niraparib as a standard maintenance therapy for epithelial ovarian cancer, and National Comprehensive Cancer Network (NCCN) category 1 preferred treatment option for the subset of patients with germline or somatic BRCA1/2 mutations.

Preclinical evidence suggests two particularly compelling reasons to investigate niraparib in cervical cancer. First, PARP inhibitors demonstrate potent radiosensitizing effects, potentially enhancing the efficacy of radiation therapy (12). Second, PARP inhibition has shown promise in preventing and reversing chemotherapy-induced peripheral neuropathy in preclinical models, addressing a common dose-limiting toxicity of standard platinum-based treatment (13). These dual mechanisms of action, radiosensitization and neuroprotection, provide a strong rationale for investigating niraparib in combination with definitive radiotherapy for LACC.

Here, we report the results of a Phase I dose-escalation study evaluating the safety, tolerability, and preliminary efficacy of niraparib administered concurrently with standard pelvic radiotherapy in patients with newly diagnosed metastatic or LACC.

## 2 Materials & methods

### 2.1 Patient population

Eligible patients had histologically confirmed FIGO 2018 stage IIIC2 (with pretreatment nodes >1 cm), IVA (with adjacent organ involvement), or IVB invasive squamous cell carcinoma or adenocarcinoma of the cervix. Key inclusion criteria were: age ≥18 years, ECOG performance status ≤1, and measurable disease by RECIST 1.1.

All patients received 3–6 cycles of platinum-based neoadjuvant chemotherapy prior to enrollment, with requirement for clinical complete or partial response within 4–12 weeks of starting protocol therapy, with any of the following seven regimens:

1. Cisplatin/paclitaxel/bevacizumab*.
2. Carboplatin/paclitaxel/bevacizumab*.
3. Cisplatin/paclitaxel.
4. Carboplatin/paclitaxel.
5. Cisplatin/topotecan.
6. Cisplatin.
7. Carboplatin.

*If bevacizumab given, 6 weeks must elapse between last dose of bevacizumab and first radiation treatment.

Adequate organ function requirements included: leukocytes ≥3,000/μL, ANC ≥1,500/μL, platelets ≥100,000/μL, creatinine ≤1.5xULN or creatinine clearance ≥30 mL/min, total bilirubin ≤1.5xULN (≤2xULN with Gilbert’s syndrome), and AST/ALT ≤2.5xULN (≤5xULN with liver metastases).

Exclusion criteria encompassed: chemotherapy, radiation, or kinase inhibitors within 4 weeks of treatment initiation; prior PARP inhibitor use; hormonal therapy within 1 week; radiation to >20% of marrow; pregnancy or lactation; severe/uncontrolled comorbidities; other malignancies within 3 years; and CNS metastases.

The study received Institutional Review Board approval, and all participants provided written informed consent.

### 2.2 Study design and treatment

The study was originally planned as an open-label, single-arm Phase I/II study. Due to slow accrual during the COVID-19 pandemic and an emerging standard of care for LACC (6, 7), the protocol was amended to include only a Phase I component. Dose escalation followed a Bayesian Optimal Interval (BOIN) design with a target DLT rate of 0.30. The BOIN design specifies pre-calculated decision boundaries: the dose is escalated when the observed DLT rate falls at or below the escalation boundary of 0.2365, de-escalated when the observed DLT rate reaches or exceeds the de-escalation boundary of 0.3585, and held at the current dose when the observed rate falls in the indeterminate interval between these boundaries. For cohorts of 3 patients, this translates to: escalate if 0 DLTs are observed, treat additional patients at the current dose if 1 DLT is observed, de-escalate if 2 or more DLTs are observed, and eliminate the dose from further study if 3 or more DLTs are observed. The maximum tolerated dose (MTD) was defined as the highest dose with a DLT rate not exceeding 30%. Calculations were performed using the R package BOIN (version 2.4). Two dose levels of niraparib (100 mg and 200 mg) were planned for evaluation; the study was designed to enroll a minimum of 3 and maximum of 9 DLT-evaluable patients at the Phase I dose level and close at establishment of a safe dose prior to initiating a Phase II expansion.

All subjects initiated niraparib at a dose of 100 mg on the first day of external beam radiation therapy. DLTs were defined per protocol (CTCAE v4.0 criteria) as any of the following events occurring during concurrent niraparib and radiotherapy: Grade ≥4 non-hematologic toxicity, Grade 3 non-hematologic toxicity lasting >3 days despite optimal supportive care (except fatigue), Grade 4 thrombocytopenia ≥14 days, Grade 3–4 thrombocytopenia with clinically significant bleeding, Grade 4 neutropenia ≥7 days or febrile neutropenia, Grade ≥3 nausea/vomiting despite optimal antiemetics, or any toxicity causing treatment delay >28 days for niraparib or >2 days for radiotherapy.

All patients were prescribed pelvic radiation using external beam radiation followed by intracavitary or interstitial brachytherapy boost consistent with clinical practice guidelines (14). 45-57.5 Gy in 1.8–2 Gy fractions of pelvic or extended field radiation were delivered, including para-aortic nodal basins if radiographically positive common iliac or para-aortic nodes were identified on pretreatment imaging. Concomitant as well as sequential nodal boost was permitted for node-positive patients. Intracavitary and/or interstitial brachytherapy was given, with niraparib not administered on brachytherapy days. Treatment planning protocol from a large multicenter prospective cohort study (EMBRACE I) was followed to establish target and organ at risk planning constraints (15).

Patients continued niraparib after radiation if they had no progression, ECOG PS 0-2, and adequate tolerability per investigator assessment. Treatment continued until progression or unacceptable toxicity.

### 2.3 Study objectives and assessments

The primary objective was to establish the maximum tolerated dose (MTD) of niraparib when administered concurrently with whole pelvic radiotherapy. Secondary objectives included acute toxicity, quality of life, and short-term primary tumor and pelvic response evaluation.

Assessments were performed weekly during concurrent treatment, including physical exam, complete blood count (CBC) with differential, adverse events per CTCAE v4.0, and quality of life using FACT-Cx questionnaire (v4.0). Post treatment assessment was performed initially at 3 months post-radiation therapy by CT or PET-CT using RECIST 1.1 criteria and at physician discretion subsequently.

## 3 Results

### 3.1 Patient characteristics and treatment delivery

A total of 4 subjects were enrolled and treated in the Phase I portion of the study before closure. Baseline characteristics are summarized in **Table 1**. One subject had locoregionally advanced disease with sigmoid and bladder invasion (FIGO IVA), while all remaining patients had distant metastases (FIGO IVB). All received neoadjuvant platinum-based chemotherapy with partial response prior to niraparib and radiotherapy treatment. Median baseline weight was 61.4 kg (range 58.5–94.8) and median baseline platelet count was 148,000/µL (range 125,000–422,000).

**Table 1:** Baseline patient characteristics and neoadjuvant therapy.

Patient ID	FIGO Stage	Neoadjuvant therapy	Baseline weight (kg)	Baseline Plt (1000/µL)	ECOG PS	Key comorbidities	Cumulative RT dose (EQD2)
NVX-1	IVB, liver metastases	6C carboplatin/paclitaxel	59.1	422	0–1	Baseline anemia	93.4
NVX-2	IVB, neck metastases	5C carboplatin/paclitaxel	63.6	135	0–1	HIV, decompensated HCV cirrhosis	82.7
NVX-3	IVB, supraclavicular, breast, axillary nodes	3C cisplatin/paclitaxel/bevacizumab, 2C cisplatin/docetaxel	94.8	125	0–1	Suspected BM injury/MDS from prior therapy	80.0
NVX-4	IVA, sigmoid and bladder invasion	6C carboplatin/paclitaxel, 1C bevacizumab	58.5	258	0–1	None reported	84.0

All subjects completed the planned pelvic radiotherapy followed by intracavitary or interstitial brachytherapy for a cumulative EQD2 >80Gy within 56 days or less from start of radiotherapy. Three patients began at the planned dose of niraparib 100 mg daily; one patient (NVX-1) inadvertently started at 200 mg daily, was subsequently corrected to 100 mg, and completed the remainder of concurrent niraparib therapy as planned. Two of the four patients (NVX-1 and NVX-4) completed concurrent niraparib therapy at the 100 mg dose level without protocol-defined DLT. NVX-4 received maintenance therapy for 5 weeks post-completion of radiation therapy, at which point treatment was discontinued after discussion of risks and benefits of continued therapy given the absence of homologous recombination deficiency or BRCA mutation.

### 3.2 Safety and dose-limiting toxicities

Treatment-related adverse events are summarized in **Table 2**. Consistent with prior safety data from large prospective trials in other disease sites, the most common low-grade toxicities were nausea, vomiting, fatigue, and hematologic abnormalities.

**Table 2:** Niraparib treatment and toxicity.

Patient ID	Niraparib dose (mg)	Niraparib duration (days)	Reason for discontinuation and toxicity details
NVX-1	200/100	56 (12 d at 200 mg, 44 d at 100 mg)	Low-grade nausea and emesis at 200 mg dose. Dose corrected to 100 mg. Completed concurrent niraparib without protocol-defined DLT. Discontinued upon completion of RT.
NVX-2	100	19 (9 d initially, 10 d after rechallenge)	Grade 2 thrombocytopenia (CTCAE v4.0). Plt from baseline 135 → 76 → 75 (held) → 71 → 77 → 80 (rechallenged) → 71 (permanently discontinued). Nadir 71. No bleeding. Investigator-initiated discontinuation; did not meet protocol DLT criteria.
NVX-3	100	2	Grade 2 thrombocytopenia (CTCAE v4.0). Plt from baseline 125 → 72 → 64 → 53 → 60 → 73 over 4 subsequent weeks. Nadir 53. No bleeding. Investigator-initiated discontinuation; did not meet protocol DLT criteria.
NVX-4	100	92	Completed concurrent and 5 weeks maintenance niraparib without protocol-defined DLT. Discontinued after discussion of risks/benefits given absence of HRD/BRCA mutation.

Two of the four treated patients (NVX-2 and NVX-3; 50%) experienced treatment-limiting grade 2 thrombocytopenia (CTCAE v4.0; platelet nadirs of 71,000/µL and 53,000/µL, respectively) and discontinued niraparib. These events did not meet the protocol definition of DLT: platelet nadirs remained above the grade 3 threshold (50,000/µL), no clinically significant bleeding occurred, no radiation therapy delays exceeding 2 days were attributable to thrombocytopenia, and in the case of NVX-2, there was no delay of more than 28 days in reinitiating niraparib. Niraparib discontinuation in both cases was at investigator discretion based on the overall clinical picture. No protocol-defined DLTs occurred in any patient.

Per the BOIN design, the observed DLT rate of 0/4 (0%) at the 100 mg dose level fell below the pre-specified escalation boundary of 0.2365, placing it in the “escalate” zone. Under normal accrual conditions, this result would have supported dose escalation to 200 mg with enrollment of a new cohort. However, the study closed before additional enrollment or dose escalation could proceed.

### 3.3 Pelvic bone marrow dosimetry

Dosimetric data for pelvic bone marrow is listed in **Table 3**. Median marrow dose was 3110 cGy for the combined patient cohort. Marrow volumes receiving at least 20 Gy (V20) and at least 40 Gy (V40) were 80.8% and 28.3%, respectively. All patients had pelvic bone marrow V20 above 75% and two patients exceeded 80%. No patients exceeded V40 above 37%.

**Table 3:** Pelvic bone marrow dosimetry.

Patient ID	BM volume (cc)	BM min dose (cGy)	BM max dose (cGy)	BM mean dose (cGy)	V20Gy (%)	V40Gy (%)
NVX-1	880	452	6260	2989	75	26
NVX-2	733	555	5607	3358	88	34
NVX-3	834	311	4829	2908	77	19
NVX-4	1023	722	4854	3232	83	34
Median	857	504	5231	3111	80	30

### 3.4 Efficacy

With a median follow-up of 30 months (range 16 - 35), all 4 patients achieved complete local (pelvic) disease control at 3, 6, and 12 months after completion of therapy. Treatment response was evaluated with CT or PET-CT at 3 months per study protocol, and thereafter imaging was performed as clinically indicated. Local control rate at 24 months for three of the patients in whom longer follow up data was available was 100%. Median progression-free survival was not reached given the low rate of observed events.

Of the cohort of 4 patients, one developed progression of liver metastases (present prior to initiating treatment) at 6-month follow-up and subsequently died of disease-related morbidity. Another patient died of non-cancer related causes and showed no clinical evidence of disease on most recent follow up. The remaining two patients remained alive with no clinical evidence of disease on most recent follow-up.

### 3.5 Quality of life

Quality of life was assessed using the FACT-Cx questionnaire, which evaluates physical, social/family, emotional, and functional well-being along with cervical cancer-specific symptoms. Scores range from 0 to 4 for each item, with higher scores indicating better quality of life.

**Figure 1:**
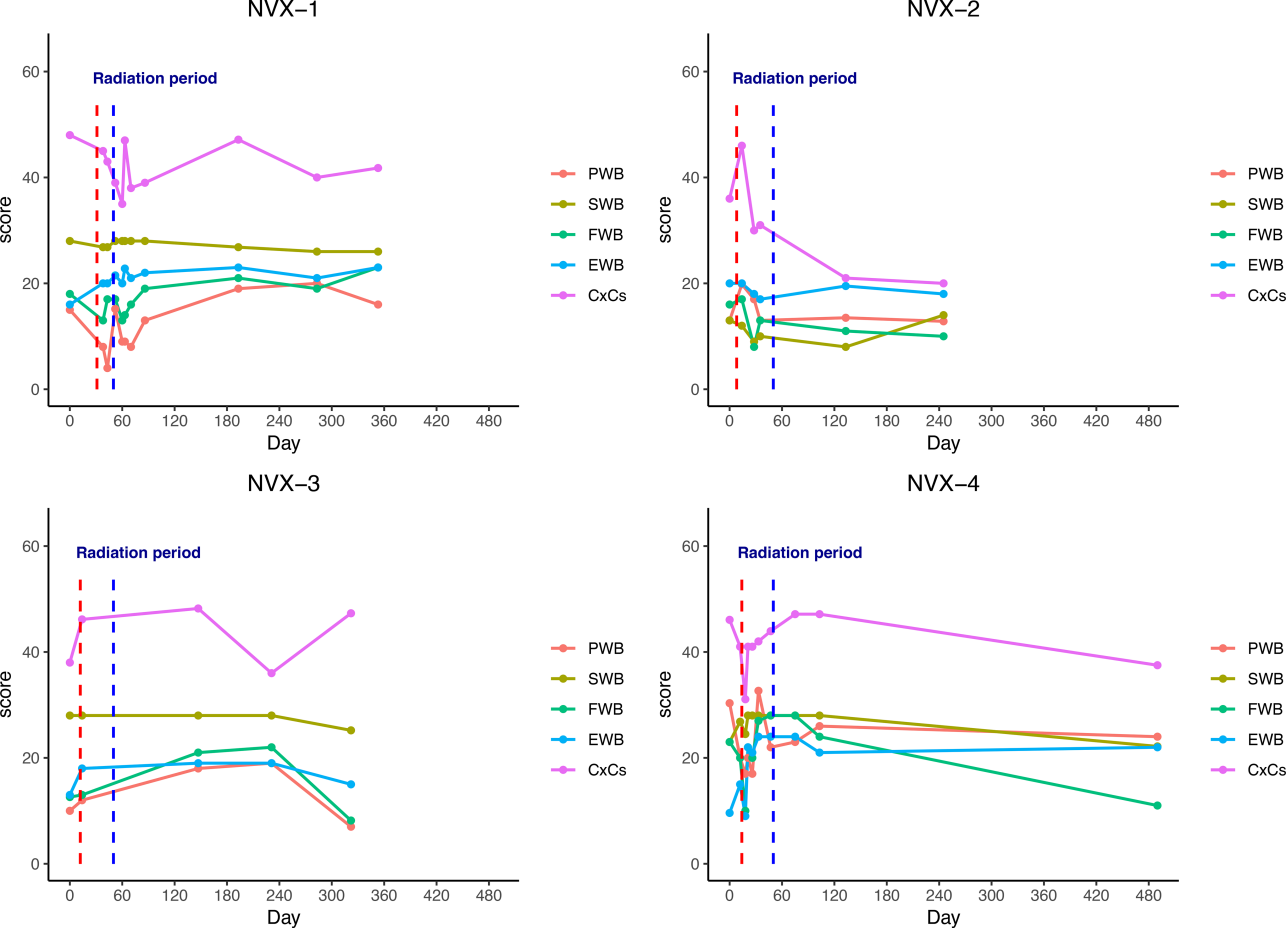
FACT-Cx quality of life scores over time for each patient (NVX1-4) over time. Color coded well-being scores for physical (PWB), social (SWB), functional (FWB), and emotional (EWB) subdomains, as well as cervical cancer specific symptoms (CxCs) are shown, with annotations indicating start and end of radiation therapy.

Mean scores for each FACT-Cx subscale at baseline and follow-up timepoints are shown in **Figure 1**. Physical and functional well-being scores declined during chemoradiation as expected, reaching a nadir at 4–5 weeks, but recovered to baseline or higher by 3 months post-treatment. Emotional well-being scores improved from baseline to 3 months. Social/family well-being remained stable throughout.

Cervical cancer symptom-specific scores, including vaginitis, pain, constipation, diarrhea and neuropathy, showed more variability between patients but overall followed a similar pattern of worsening acutely during treatment and normalizing by 3 months. No unexpected late toxicities or persistent detriments in quality of life were observed with the addition of niraparib.

## 4 Discussion

To our knowledge, this is the first clinical trial to evaluate a PARP inhibitor concurrently with definitive radiation in metastatic or LACC. The primary objective was to establish the recommended MTD of niraparib in combination with standard pelvic radiotherapy for further investigation. Although the study was terminated early, the preliminary results are encouraging and hypothesis-generating. At the 100 mg dose level, 2 of 4 patients (NVX-1 and NVX-4) completed the planned concurrent niraparib without any DLTs, demonstrating the feasibility of this combination in a cohort of patients previously treated with systemic chemotherapy.

### 4.1 Safety and dose-limiting toxicities

The hematologic toxicity observed in our cohort prompts a more detailed exploration of the risks of niraparib therapy. A combined analysis of toxicity data from three large phase III studies of niraparib for ovarian cancer found that apart from low grade nausea (74% overall; 3% grade 3+) thrombocytopenia was the most common adverse effect (61% overall; 34% grade 3+), and far higher with niraparib compared to other PARPi (16). Moreover, thrombocytopenia onset was rapid, with NOVA trial data showing platelet drop beginning near week 2 and nadir by approximately week 3 of therapy, and a large majority of patients experienced dose interruptions or reductions due to toxicity (80% and 73%, respectively) (10, 17). Despite the high event rate, thrombocytopenia was transient with return to near baseline noted after the first month; less than 1% of events occurred after 3 months, with no cumulative toxicity and low rates of MDS (1.4%) (10). Furthermore, no negative quality of life impact from hematologic toxicity was observed from validated patient reported outcomes data (18).

A closer evaluation of the patients with DLT in our study suggests that patient-specific factors likely played a significant role in the observed thrombocytopenia. Descriptive comparison of the two patients who discontinued (NVX-2 and NVX-3) versus the two who completed concurrent therapy (NVX-1 and NVX-4) is informative, though we emphasize that no statistical inference is appropriate given the sample size of 4 patients.

NVX-2 had HIV and decompensated HCV cirrhosis. The borderline-low baseline platelet count of 135,000/µL is consistent with portal hypertension and splenic sequestration commonly seen in cirrhotic patients. Additionally, niraparib undergoes hepatic metabolism via carboxylesterase-catalyzed amide hydrolysis (19), and impaired hepatic function may have altered drug exposure. Platelet counts dropped from 135,000 to a nadir of 71,000/µL (grade 2 per CTCAE v4.0) by day 9 of therapy. After a 4-week drug holiday, niraparib was rechallenged but again produced thrombocytopenia, prompting permanent discontinuation at investigator discretion. No clinically significant bleeding occurred, and no radiation therapy delays exceeding 2 days were attributable to this toxicity.

NVX-3 similarly had a borderline low baseline platelet count of 125,000/µL and received only 2 doses of concurrent niraparib before platelet count fell to 72,000/µL. The remarkably rapid onset, within 2 days of the first dose, is atypical for niraparib-induced thrombocytopenia, where FDA Adverse Event Reporting System data has shown a mean onset of 21 days (IQR 9–74) (20). This patient was subsequently found to have persistent pancytopenia throughout follow-up, raising clinical suspicion for bone marrow injury or myelodysplastic syndrome secondary to prior neoadjuvant chemotherapy. The nadir of 53,000/µL corresponds to grade 2 thrombocytopenia per CTCAE v4.0.

Both patients who discontinued niraparib had baseline platelet counts below 150,000/µL, consistent with risk-factor data from a NOVA trial analysis that identified baseline weight and baseline platelet count as significant predictors of grade 3+ thrombocytopenia (16% vs 45% for weights of ≥77 kg and ≤58 kg, respectively; 20% vs 42% for platelet counts of ≥273,000/µL and ≤180,000/µL, respectively) (17). In contrast, the two patients who completed concurrent therapy (NVX-1 and NVX-4) had baseline platelet counts of 422,000/µL and 258,000/µL, respectively, well above identified risk thresholds. Additionally, 3 of 4 patients in our cohort were near the 58 kg weight threshold, representing a population at higher baseline risk for hematologic events. These observations are hypothesis-generating and should not be interpreted as predictive; adequately powered studies with formal risk-factor analysis are needed.

Future studies of PARP inhibitors with radiation should consider more conservative inclusion criteria with attention to such risk factors to limit treatment-limiting toxicities and ensure optimal patient selection. Requiring baseline platelet counts above 150,000/µL and screening for hepatic dysfunction may represent reasonable modifications for future trial design.

Apart from the above noted thrombocytopenia, niraparib was well tolerated at the 100 mg starting dose. Low grade toxicities of nausea, vomiting, and fatigue were primarily observed in one patient (NVX-1) who inadvertently began taking 200 mg niraparib due to misunderstanding of dosage instructions. Upon dose correction, these symptoms improved and did not lead to DLT.

### 4.2 Radiation therapy

Radiation delivery and technique in our study followed well established standards and all patients met dosimetric constraints from RTOG 0418, where a secondary analysis found median dose to bone marrow (median > 34.2 Gy) and volume receiving 40 Gy (V40 > 37%) as predictive for grade 2 and higher hematologic toxicity (21). Additionally, the very short observed time frame of 2 and 9 days after start of therapy is not consistent with pelvic radiation induced hematologic toxicity, where anemia and leukopenia is more commonly seen, and typically follows a gradual decline with nadir of approximately 34–35 days (22). Given these observations it is unlikely that radiation played a significant role in the observed thrombocytopenia in our study. However, it should be noted that modern paradigms of neoadjuvant chemotherapy show higher rates of hematologic toxicity, with neoadjuvant chemotherapy followed by definitive chemoradiation showing thrombocytopenia rates of 50% (16% grade 3 and above) in one phase III study (23), and even concurrent immunotherapy more than doubled rates of grade 3 and higher thrombocytopenia in the landmark KEYNOTE-A18 study (5% vs 2% in placebo arm), although the study was not powered to assess differences in these results (6). Newer data suggests that bone marrow volumes receiving lower doses of 5, 10, and 20 Gy may be of greater significance for predicting hematologic toxicities (24–27), and more conservative dose constraints may represent an increasingly important consideration in improving outcomes and reducing toxicity.

### 4.3 Efficacy

All patients achieved pelvic disease control at 12 months, and at 24 months for the three patients in whom longer follow up data was available. Distant failure and disease-specific mortality occurred in one patient (NVX-1), with progression of liver metastases present prior to initiating treatment. This compares favorably to historical local control rates ranging 67 – 92% with standard chemoradiation in LACC (5, 15). The small size of this study precludes drawing meaningful conclusions regarding treatment efficacy; ultimately, larger studies with longer follow-up are needed to determine if niraparib can improve locoregional control rates and survival outcomes when integrated with definitive radiotherapy. As an exploratory hypothesis, the observed 100% pelvic disease control in this cohort suggests that concurrent PARP inhibition may augment locoregional disease control, but this must be interpreted with caution given the small sample size and absence of a comparator arm.

### 4.4 Quality of life

Patient-reported quality of life outcomes suggest that concurrent and maintenance niraparib with definitive chemoradiation is overall well-tolerated without compromising quality of life outcomes expected for standard treatment. Our study observed the expected pattern of acute worsening during radiation followed by recovery at 3 months. Emotional well-being improved over the course of treatment, perhaps reflecting psychological benefit from the patients’ hope and satisfaction with response to a novel therapeutic approach for their high-risk disease. These findings are consistent with previously reported quality of life outcomes in this cohort of patients from a safety net hospital (28).

### 4.5 Limitations

The main limitations of this study are the small sample size (n=4) and short follow-up duration due to early termination. With only 4 patients treated at a single dose level, the BOIN design could not complete its intended dose-finding algorithm, and the MTD was not formally established. The efficacy and quality of life results are considered exploratory and hypothesis-generating rather than conclusive. Only the 100 mg dose of niraparib was evaluated before closure, so the optimal dose and schedule to combine with chemoradiation remains to be determined. Descriptive comparisons of patient-level risk factors for thrombocytopenia are informative but should not be interpreted as formal predictive analyses. Future randomized trials with adequate power are needed to assess the true benefits and risks compared to standard treatment.

### 4.6 Conclusion and future directions

Results from this Phase I study suggest that niraparib at 100 mg doses can be safely administered concurrently with pelvic radiotherapy for metastatic or LACC in appropriately selected patients. No protocol-defined DLTs were observed, and the treatment-limiting toxicity of grade 2 thrombocytopenia appeared driven by patient-specific risk factors amenable to screening. Moving forward, it may be possible to identify predictive biomarkers that could be used to select patients for whom this treatment combination may be more safely utilized. However, the small number of subjects enrolled in this trial precluded the identification of such biomarkers. Correlative analyses of tumor DNA repair deficiency (e.g., homologous recombination status), immune microenvironment, and circulating cytokines may help elucidate the biological mechanisms and optimize patient selection for PARP inhibitor combinations in future trials.

The standard-of-care landscape for LACC has evolved substantially since this trial was initiated. The KEYNOTE-A18 trial demonstrated improved progression-free survival with the addition of pembrolizumab to concurrent chemoradiation followed by adjuvant pembrolizumab (6), establishing immunotherapy-chemoradiation as a new standard for eligible patients. Similarly, the INTERLACE trial supports the use of neoadjuvant chemotherapy prior to chemoradiation (7). In this evolving treatment paradigm, PARP inhibition may serve a complementary role. Preclinical data suggest that PARP inhibitors activate the cGAS-STING innate immune signaling pathway and increase tumor mutational burden, potentially enhancing the immunogenicity of the tumor microenvironment and thereby augmenting the efficacy of immune checkpoint inhibitors (29). Combining PARP inhibition with immunotherapy-radiation regimens represents a rational multi-modality strategy warranting investigation.

Several clinical design strategies merit consideration for future trials. These include concurrent PARP inhibitor administration during chemoimmunotherapy-radiation, sequential PARP inhibitor maintenance following immunotherapy-chemoradiation, and dose-optimization studies in patients pre-selected for favorable risk profiles (e.g., baseline platelet count >150,000/µL, absence of significant hepatic dysfunction, and body weight >77 kg). Bone marrow-sparing radiation techniques with attention to low-dose volumes (V5, V10, V20) may also mitigate hematologic toxicity when combining these agents. As an exploratory hypothesis, we propose that concurrent PARP inhibition may enhance locoregional disease control through radiosensitization mechanisms that are potentially independent of homologous recombination deficiency status, and that these benefits may complement the systemic disease control afforded by immune checkpoint inhibitors. This hypothesis requires testing in adequately powered randomized trials.

Importantly, no unexpected adverse events were identified in our study subjects. Preliminary efficacy and quality of life outcomes are encouraging but require validation in larger trials. Findings from our study suggest that patient selection and toxicity profiles of novel treatments should be given careful consideration to ensure optimal benefit and minimize harm.

## Data Availability

The raw data supporting the conclusions of this article will be made available by the authors, without undue reservation.
